# 3D Printing of Polymeric Multi-Layer Micro-Perforated Panels for Tunable Wideband Sound Absorption

**DOI:** 10.3390/polym12020360

**Published:** 2020-02-06

**Authors:** Wenjing Yang, Xueyu Bai, Wei Zhu, Raj Kiran, Jia An, Chee Kai Chua, Kun Zhou

**Affiliations:** 1 Singapore Center for 3D Printing, School of Mechanical and Aerospace Engineering, Nanyang Technological University, 50 Nanyang Avenue, Singapore 639798, Singapore; wyang011@e.ntu.edu.sg (W.Y.); xueyu001@e.ntu.edu.sg (X.B.); zhu.wei@ntu.edu.sg (W.Z.); raj005@e.ntu.edu.sg (R.K.); anjia@ntu.edu.sg (J.A.); 2 Engineering Product Development Pillar, Singapore University of Technology and Design, 8 Somapah Rd, Singapore 487372, Singapore; cheekai_chua@sutd.edu.sg

**Keywords:** 3D printing, selective laser sintering, micro-perforated panel, acoustic absorption, multi-layer structure

## Abstract

The increasing concern about noise pollution has accelerated the development of acoustic absorption and damping devices. However, conventional subtractive manufacturing can only fabricate absorption devices with simple geometric shapes that are unable to achieve high absorption coefficients in wide frequency ranges. In this paper, novel multi-layer micro-perforated panels (MPPs) with tunable wideband absorption are designed and fabricated by 3D printing or additive manufacturing. Selective laser sintering (SLS), which is an advanced powder-based 3D printing technique, is newly introduced for MPP manufacturing with polyamide 12 as the feedstock. The acoustic performances of the MPPs are investigated by theoretical, numerical, and experimental methods. The results reveal that the absorption frequency bandwidths of the structures are wider than those of conventional single-layer MPPs, while the absorption coefficients remain comparable or even higher. The frequency ranges can be tuned by varying the air gap distances and the inter-layer distances. Furthermore, an optimization method is introduced for structural designs of MPPs with the most effective sound absorption performances in the target frequency ranges. This study reveals the potential of 3D printing to fabricate acoustic devices with effective tunable sound absorption behaviors and provides an optimization method for future structural design of the wideband sound absorption devices.

## 1. Introduction

Micro-perforated panels (MPPs) are the next-generation solutions for noise reduction with the potential of achieving high absorption coefficients for wideband sound absorption. The MPP was first studied by Maa for sound absorption research [1,2,3]. Since then, the applications of MPPs have been broadened to room acoustic conditions, duct silencing, acoustic window systems, and noise barriers [4,5,6]. An MPP system consists of a thin panel with perforations, a back wall, and an air gap. The acoustic resistance is provided by the lattice of perforations with the diameter in a sub-millimeter range on the thin panel. The panel is backed by a rigid wall, which is parallel to the panel. The air gap between the wall and the panel generates an acoustic stiffness dependent on the depth of the air gap. The entire MPP system provides resonant absorption of sound. It should be noted that the absorption effect is independent of the manufacturing materials of the panel [1,2].

Innovative designs of MPPs for wide bandwidth absorption with high efficiency have been studied extensively. The shape of the panel perforations has been of great interest for sound absorption applications. For example, the micro-slit panels instead of circular-perforated panels were proposed and studied in detail [7,8]. In addition, Ning et al. investigated the acoustic impedance and absorption coefficients of the MPPs with triangle-shaped, square-shaped, and irregular circle-shaped perforations [9]. Besides the perforation shapes, the effects of the air gap distances were also investigated. The theories and simulations of MPPs with two different air gap depths were provided by Sakagami et al. [10]. Wang et al. conducted acoustic experiments on a single MPP panel backed by three different air gap depths and reported the high absorption efficiency of such MPP structures [11]. Wang et al. also altered and enhanced the sound absorption efficiency by modifying the air gap to a trapezoidal cavity [12]. Furthermore, partitioning the air cavity and subdividing cavities were also concluded to be effective to improve the acoustic performance of the MPPs. The popular partitions were honeycomb cavities and L-shape cavities [13,14,15]. Another appealing solution to broaden the effective bandwidth is the multi-layer MPP or the multi-leaf absorber. Maa proposed a double-layer MPP backed with a rigid wall in his early research [1,2]. These studies were later extended to the multi-layer MPP structures [16,17]. An alternative multi-layer structure named a multi-leaf MPP space absorber was also developed and the structure consisted of multiple panels and air gaps in-between without the rigid back wall [18,19].

Since the acoustic absorption performance is independent of the manufacturing material of MPPs, for the convenience of the perforation fabrication, metal or steel have been mainly used to produce the MPP panels for experimental validations. The perforations are produced by costly manufacturing methods including laser cutting, micro punch, etching, etc. on the panels, which generally constrain the widespread applications of the MPPs. In light of these facts, novel techniques and materials are under development to reduce the cost and speed up the fabrication process. The micro-slit or micro-slot MPP panels with perforations in easy-drilling shapes were studied to reduce the complexity of fabrication [7,8,20]. An infiltration method was introduced by Cobo and Espinosa to reduce the manufacturing cost [21]. However, the process was time-consuming and also resulted in low accuracy. The macro-perforated panels backed by meshes [22] and parallel-perforated ceramic materials [23] were also proposed to reduce the manufacturing difficulty. The Micro-Electro-Mechanical Systems were utilized to fabricate ultra-micro perforated MPPs for acoustic impedance and an absorption improvement [24]. Recently, Liu et al. [25,26] introduced the 3D printing technique for high-precision and fast fabrication of MPPs as a pioneer. The Stereolithography Apparatus (SLA) was employed for MPP manufacturing to study the effects of the perforation angle and air gap distance on sound absorption [25]. Polyjet was applied to fabricate MPPs with different perforation ratios at a high-frequency range (1500–5500 Hz) and a high accuracy of the micro-circular perforations with a diameter of 0.6 mm was reported [26]. The use of polymer materials in these studies suggests that polymeric MPPs are much lighter and more cost-efficient as compared to the conventional metal MPPs.

The 3D printing techniques are ideal solutions to complex structures [27,28,29], but their application to acoustic structures is still at an early stage with very limited literature. Selective Laser Sintering (SLS), which is a powder-bed-fusion 3D printing process [30,31,32,33], has the potential [34,35,36] to rapidly fabricate the MPP structures by using various polymer materials in a single step without tooling or molding processes [37,38]. It fabricates 3D objects by using a CO_2_ laser beam to sinter thermoplastic polymer powders by using each layer. The cross sections of the parts are selectively sintered, while the un-sintered powders are served as supports and are highly recyclable [37]. A wide range of materials can be processed by SLS. One major advantage of SLS over other 3D printing techniques (e.g., Polyjet and SLA) is the exclusion of supporting structures to build complex objects such as hollow and overhang structures [39]. This support-free feature is ideal for fabricating complex hollow structures with multiple layers where conventional methods would require post-fabrication assembly to integrate individual components into multiple layers. The utilization of 3D printing techniques can facilitate the proof of concept of the designed acoustic structures by rapid manufacturing of prototypes [40]. It has great potential to expand for mass manufacturing in acoustic areas.

In this study, multi-layer MPPs for tunable wideband sound absorptions were designed and manufactured. The SLS technology has been introduced for the first time. To the best of the authors’ knowledge, this technology manufactures polymeric multi-layer structures with perforations in a single step without the need of subtractive manufacturing and multi-structure assembly. The parameters of the structures in this study were designed for damping major traffic noises at a low frequency of around 1000 Hz [41]. The effects of geometric parameters on the absorption coefficient and the frequency range were predicted by theories and numerical simulations, and were further evaluated by the experiments. Furthermore, an optimization method by maximizing the area under the sound absorption curve at the target frequency range was introduced to design the structural parameters of the effective, acoustic absorption devices. The optimization methods were validated experimentally by the optimized multi-layer structures that were rapidly manufactured by SLS.

## 2. Materials and Methods

### 2.1. Theoretical and Numerical Methods

**Figure 1:**
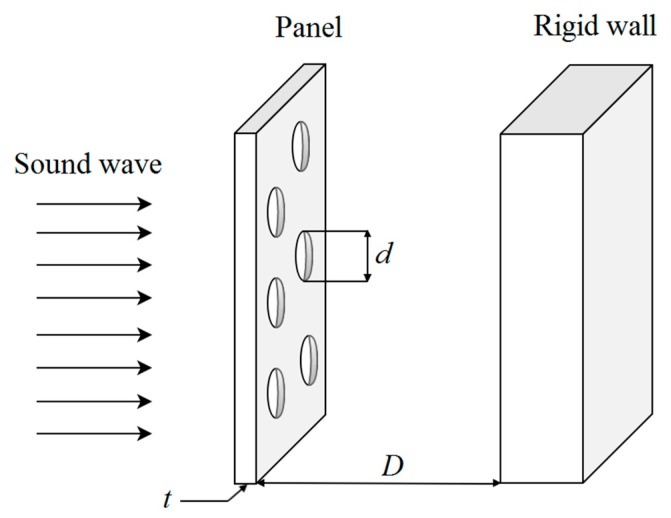
The schematic diagram of an MPP, which consists of a thin panel, an air gap, and a rigid wall.

In this study, the acoustic absorption of the MPP is predicted by Maa’s theory [1,2,3]. The theory summarizes that the absorption coefficient and the effective frequency range are independent of materials but dependent on the geometric dimensions of the MPP. The thickness of the panel *t*, the perforation diameter *d*, the perforation ratio *p*, and the air gap distance *D* are the geometric variables determining the absorption efficiency at a given frequency. Figure 1 shows the schematic configuration of a single-layer MPP and the parameters *t*, *d*, and *D*.

Acoustic waves are small harmonic variations of pressure in a fluid, which are superimposed on a background pressure. The fundamental equation governing this sound wave propagation in a short tube is shown below [3,42,43].

(1) $$\frac{\partial^{2}v}{\partial x^{2}}+\frac{\partial^{2}v}{\partial y^{2}}-\frac{j\omega \rho_{0}}{\eta }v=\frac{1}{\eta }\frac{\Delta p}{l},$$

where *v* is the particle velocity, *ρ*_0_ and *η* are the density and viscosity of air, respectively, *ω* is the angular frequency, Δ*p* is the sound pressure difference between the two ends of the tube, and *l* is the length of the tube’s unit element. The particle velocity *v* is obtained analytically and is equal to zero at the tube walls.

The specific acoustic impedance *Z*_total_ is defined as the ratio of the sound pressure difference to the average velocity.

(2) $$Z_{\mathrm{total}}=\frac{\Delta p}{\bar{v}}.$$

An approximate theoretical solution to the specific acoustic impedance of the MPP with circular pores was introduced by Maa [1,2,3]. The total normalized specific acoustic impedance *Z*_total_ is calculated by summing up the acoustic impedance of the panel layer and the air gap. The acoustic impedance of the air gap is calculated by using the equation below.

(3) $$Z_{D}=-j\cot\frac{2\pi fD}{C_{0}},$$

where the constant *C*_0_ is the speed of sound in the air and *f* is the frequency. The acoustic impedance of the panel is derived by using a panel model that consists of an array of tubes and is expressed by the equation below [1].

(4) $$Z_{M}=\frac{32\mu t}{pc_{0}d^{2}}(\sqrt{1+\frac{q^{2}}{32}}+\frac{\sqrt{2}q}{8}\frac{d}{t})+\frac{j\omega t}{pc_{0}}(1+\frac{1}{\sqrt{9+\frac{q^{2}}{2}}}+0.85\frac{d}{t}),$$

where the perforation constant *q* is shown below.

(5) $$q=\sqrt{\frac{\omega }{\mu }}\frac{d}{2},$$

The constant *µ* is the coefficient of the kinematic viscosity of air. The sound absorption coefficient *α* of the MPP is shown in the equation below.

(6) α=4Re(Ztotal)[1+Re(Ztotal)]2+[Im(Ztotal)]2,

where Re(*Z*) and Im(*Z*) represent the real part and the imaginary part of the impedance, respectively.

Maa’s theory simplifies the multi-layer MPPs as a series-parallel connection system of the acoustic elements (MPP and cavity) [1]. The acoustic impedance of each series-connection subsection is first calculated upon which the total acoustic impedance can be obtained by applying the parallel-connection rule. The acoustic impedance at the input of the *n*^th^ series-connection MPP-cavity layer is given by the equation below.

(7) $$Z(n)=Z_{M}(n)+Z_{C}(n),$$

where *n* = 1, 2, …, *N* − 1 with *N* being the total number of the layers. *Z_M_*(*n*) and *Z_C_*(*n*) are the acoustic impedance of the MPP and cavity of the *n*^th^ layer, wherein the subscripts *M* and *C* represent the initials of the MPP and cavity, respectively. The acoustic impedance *Z_C_*(*n*) of the *n*^th^ cavity is related to the acoustic impedance *Z*(*n* + 1) of the (*n* + 1)^th^ layer, which is shown below.

(8) $$\frac{1}{Z_{C}(n)}=\frac{1}{Z_{CS}(n)}+\frac{1}{Z(n+1)},$$

where the acoustic impedance of the single cavity of the *n*^th^ layer *Z_CS_*(*n*) is the same as *Z_D_* in Equation (3). The calculation of the acoustic impedance of the MPP of the *n*^th^ layer *Z_M_*(*n*) is identical to *Z_M_* in Equation (4).

In the Maa’s equivalent circuit model for multi-layer MPPs, the panel-to-panel and panel-to-air gap interactions were simplified. To this end, 3D finite element method-based (FEM) numerical simulations were carried out to solve the governing Equation (1) using the pre-set module “Thermo-acoustics” in “COMSOL Multiphysics” software. The velocity and pressure field in the MPPs was calculated, and the surface impedance was extracted through Equation (2).

Given the periodicity of the pores, only a quarter of the unit cell containing one pore and the corresponding incidence part of the back cavity was sufficient to model the whole MPP structure. In the finite element (FE) model, only the fluid region (air) with symmetrical boundaries was created. Sound-hard boundaries were imposed on the interfaces between air and the MPP plane because the fluid-solid coupling effect was ignored. In order to fully capture the influence of viscous boundary layers, the smallest mesh size inside the micro-perforations adjacent to the perforation walls was smaller than the thickness of the viscous boundary layer at the highest frequency. Moreover, hexahedron elements were used. A mesh sensitivity study indicated that the element sizes could effectively balance the simulation accuracy and efficiency and, thus, the calculation convergence was guaranteed. With a normal incidence plane wave with amplitude 1 Pa impinging on the front surface of the MPP, the velocity and pressure field were determined accordingly.

### 2.2. Experimental Method

**Figure 2:**
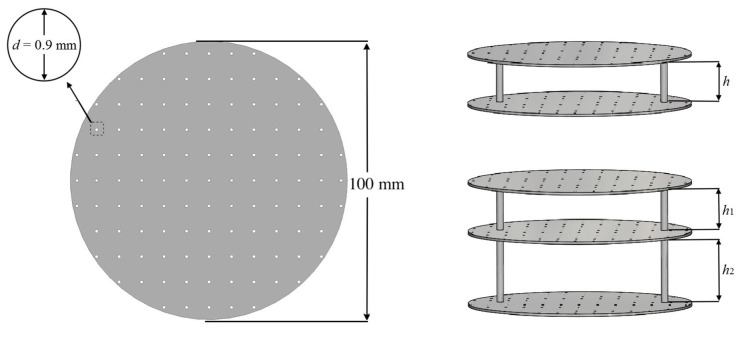
The schematic demonstration of the single-layer, double-layer, and triple-layer MPPs.

Multi-layer structures with single, double, and triple layers of identical panels were designed for characterising the sound absorption capability of MPPs. Figure 2 shows the geometric configurations of the structures. The geometric dimensions were designed to match the resolution of SLS [37] and to provide tuneable acoustic impedances and sound absorption coefficients within the target frequency range of 200 to 1600 Hz.

The panel thickness *t*, perforation diameter *d*, perforation ratio *p*, and panel diameter of the panels were kept at 1 mm, 0.9 mm, 1.0 %, and 100 mm, respectively. The arrangement was made such that the inter-layer distances *h* (double-layer), *h*_1_, and *h*_2_ (triple-layer) in Figure 2 could vary from 20 mm to 40 mm to quantify the effects of different *h* on the effective range of *f* and the absorption coefficient *α*. The geometric dimensions are shown in Table 1. All of the structures were manufactured in one batch by an SLS P395 machine (EOS, Krailling, Germany) with a CO_2_ laser (wavelength: 10.6 µm, laser power: up to 50W). PA12 (PA2200 from EOS GmbH, Krailling, Germany), which is one of the commercial materials with advantages of a high melting flow rate, low melting temperature, low glass transition temperature, and a low degree of crystallization temperature [44], was used as the feedstock. The mechanical properties and viscosity of laser-sintered PA12 are shown in Table 2. The commercial printing parameters for PA12 from EOS P395 system including laser scanning speed of 4000 mm/s, laser power of 40 W, layer thickness of 0.12 mm, and hatching space of 0.3 mm were followed.

**Figure 3:**
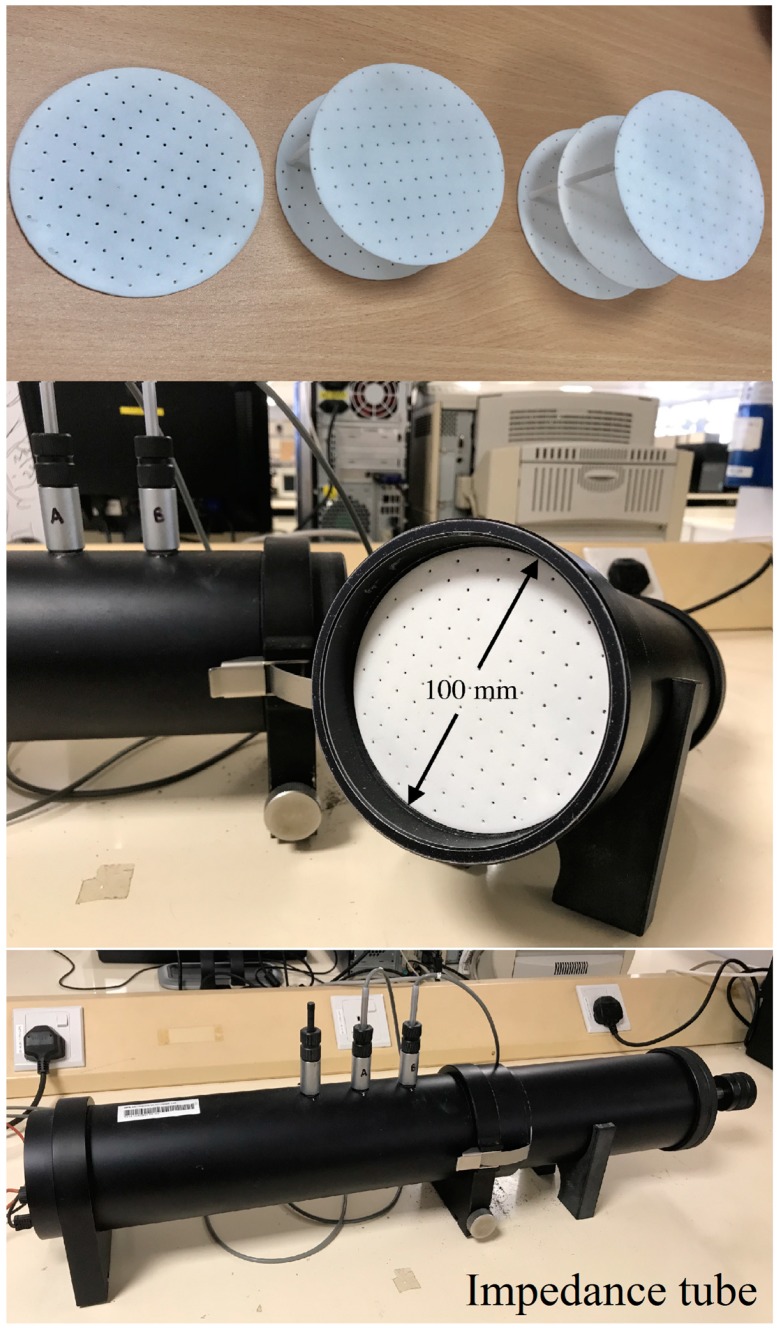
3D printed samples of MPPs and the setup of the acoustic absorption test.

**Figure 4:**
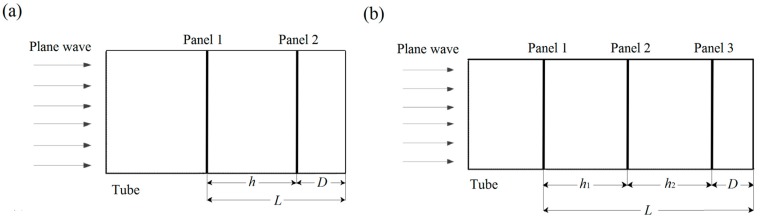
The schematic demonstration of the multi-layer MPPs: (**a**) a double-layer MPP and (**b**) a triple-layer MPP in the impedance tube.

The typical samples of 3D-printed MPPs and the experimental setup are shown in Figure 3. A two-microphone impedance method using impedance tube type 4206 (Brüel and Kjær Sound and Vibration, Nærum, Denmark) was used to measure the sound absorption of the specimens. The impedance tube with an inner diameter of 100 mm measured the frequency range from 50 to 1600 Hz. The sound absorption coefficients were calculated by a transfer function method [45]. The air gap distance *D* of MPPs could be adjusted to tune the absorption frequency range. Figure 4 shows the schematic configurations of the double-layer and triple-layer MPP structures including the air gap in the tube. The total length *L* was defined as the length of the MPP system including the thickness of the panels, the inter-layer distances, and the air gap distances.

## 3. Results and Discussion

### 3.1. Effect of the Number of Layers

**Figure 5:**
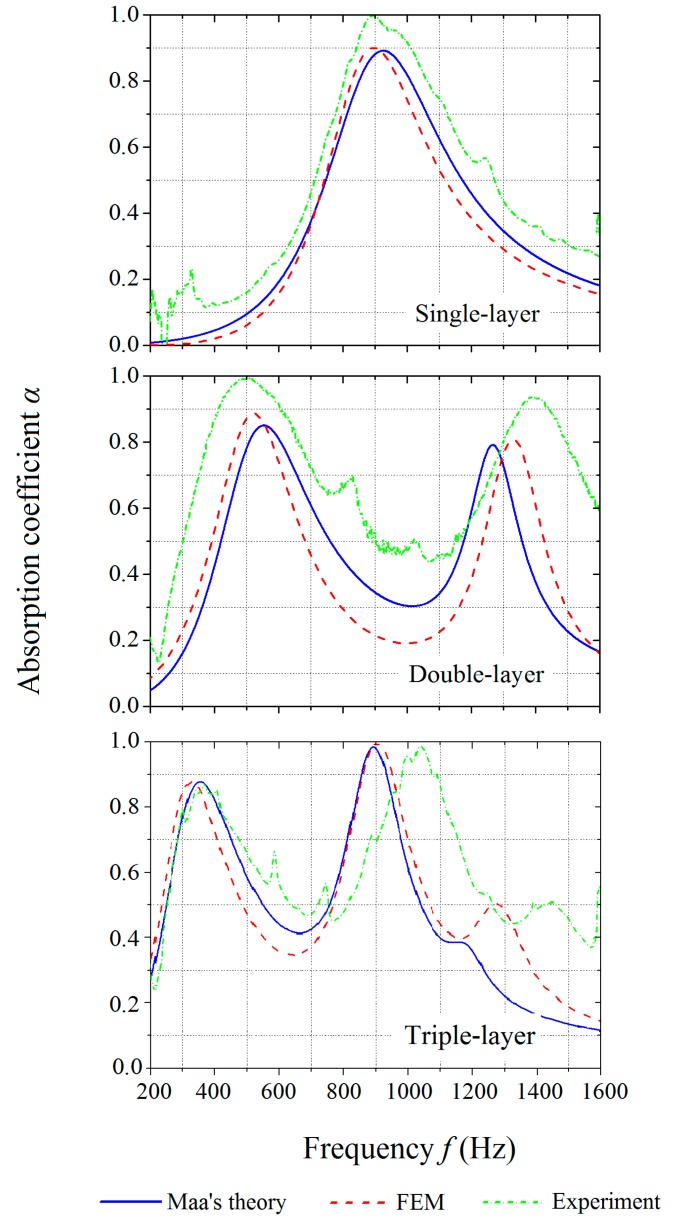
Comparison of the absorption coefficients of the single-layer, double-layer, and triple-layer MPPs obtained by the theoretical predictions, numerical simulations, and experiments.

Figure 5 shows the comparison of the absorption coefficients of MPP_1, MPP_2, and MPP_6. The air gap between the rigid wall and the structures was kept at 20 mm. The frequency range was restricted to 200–1600 Hz so that the noise could be filtered out at low frequencies (below 200 Hz).

The numerical simulation results have a reasonable correlation with values from the theoretical predictions, which indicate the acceptable prediction capability of the developed FE model. The experimental results agree well with the theoretical and numerical results. Both of the double-layer and triple-layer structures tune the peaks of the absorption curves to different frequencies so that the frequency bandwidth is broadened with the values of the absorption coefficient kept at 0.8 or above. An increase in the number of layers is able to increase the number of the peaks on the absorption coefficient curves and shifted the effective frequency range. The absorption curves of the double-layer MPP and the triple-layer MPP observe two and three peaks, respectively, for the given geometric parameters.

The experimental results with unpredicted noises and side peaks on the absorption curves show wider frequency bandwidths and higher peak values of the MPPs than the theoretical and numerical results. The discrepancies are more clear at the second peak on the curves of the double-layer and triple-layer MPPs, respectively. It is observed that the second peak on the curve of the experimental results of the triple-layer MPP has slightly shifted to the higher frequency range as compared to those predicted by the theory and numerical simulations.

**Figure 6:**
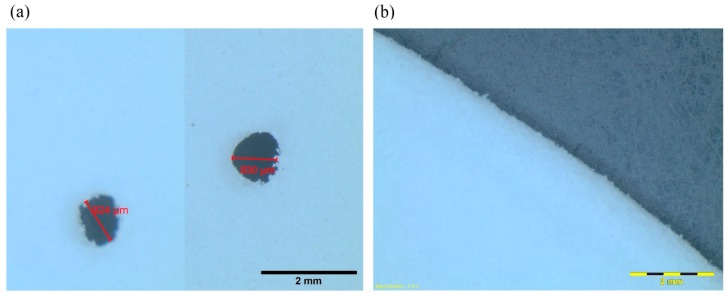
The microscope images of a printed panel: (**a**) two perforations in imperfect circular shapes and (**b**) a part of the unsmooth rim with grains.

The discrepancies could be attributed to the following reasons: (i) the perforations in the printed structures were not perfectly circular in shape (as shown in Figure 6a); (ii) since the diameters of the perforations were all smaller than 1 mm, they were not consistent and not precisely equal for the designed values of 0.9 mm; (iii) the unsmooth rims of the panels resulted in air spaces between the panels and the duct wall (as shown in Figure 6b), which affected the wave flow during the acoustic tests but were not taken into account by the theory or the FE model; (iv) the peripheral warpage was caused by the thermal gradient during the SLS printing process, which might have resulted in irregular gaps between the circular panel and the tube wall [46]; (v) the pillars between the panels were ignored by the theory and the FE model; and (vi) the sound speed *C*_0_ in the air and the coefficient of the kinematic viscosity of air *µ* were assumed to be constant in the theory and FEM simulations but could have varied due to the temperature change in experiments. These previously mentioned causes of the discrepancies among the theory, FEM, and experiment results are also valid in the subsequent studies of tuning the absorption frequency ranges and optimizing the structural designs.

### 3.2. Effect of the Air Gap Distance

**Figure 7:**
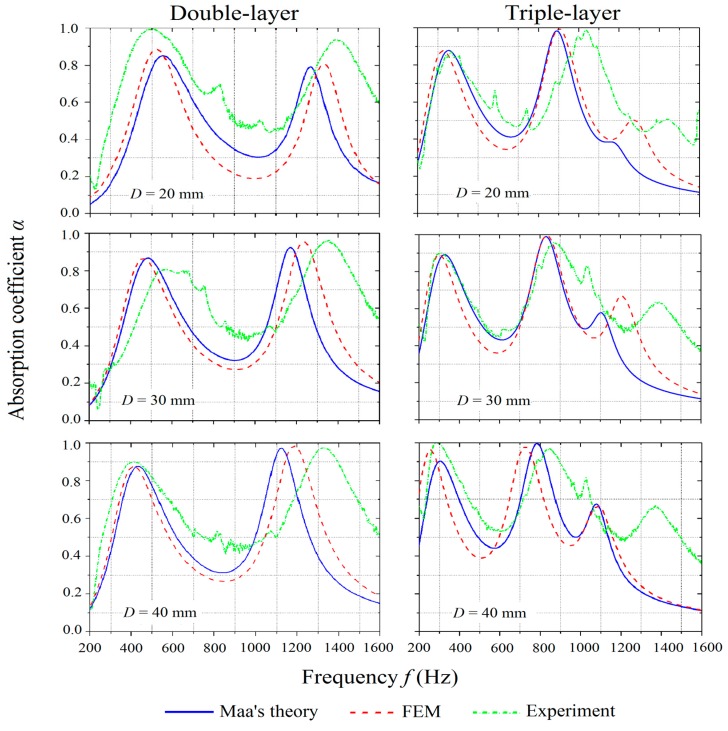
Comparison of absorption coefficients of MPPs with varying air gap distances obtained by the theoretical predictions, numerical simulations, and experiments.

To study the effect of the air gap distances, MPP_2 and MPP_6 were considered with air gap distances of 20 mm, 30 mm, and 40 mm and the results are shown in Figure 7. The experimental results show similar trends as predicted by the theoretical and numerical results with varying air gap distances. However, the experimental results dictate a wider frequency bandwidth and a higher overall absorption coefficient.

For both double-layer and triple-layer structures, the increase of the air gap distances results in the left shift of the peaks. Thus, this tunes the effective absorption to a lower frequency range, which is similar to the single-layer structure that has been studied by Maa [1,2,3]. The width of the frequency range and the peak values, however, do not change significantly. It is interesting to note that, at the lower frequencies, the absorption coefficient almost remains constant while the air gap distances increase. The effect can be explained by considering the perforation panels as the mass while the air column as an acoustic spring. The increase of the air gap distance reduces the stiffness of the spring, which causes the resonance of the spring and the mass system to shift to a lower frequency range. For the triple-layer MPP, as the air gap distance increases, the absorption coefficient corresponding to the third peak on the curve increases sharply.

### 3.3. Effect of the Inter-Layer Distance

**Figure 8:**
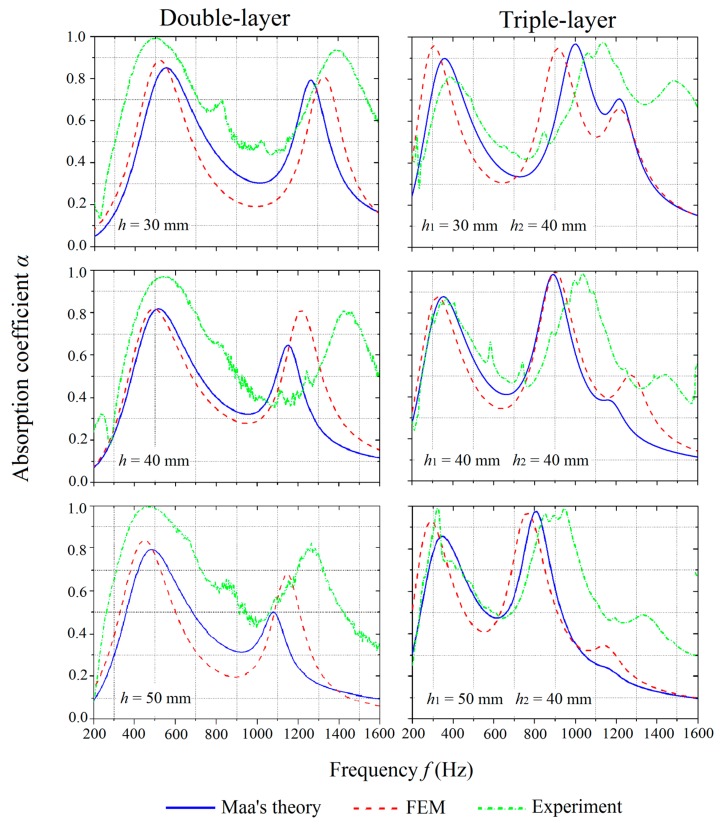
Comparison of absorption coefficients of the MPPs with varying inter-layer distances obtained by the theoretical predictions, numerical simulations, and experiments.

To study the effect of the inter-layer distance on the absorption coefficient, MPP_2, MPP_3, and MPP_4 were compared under the category of double-layer MPPs while MPP_5, MPP_6, and MPP_7 were compared under the category of triple-layer MPPs, as shown in Figure 8. The air gap distances were kept at 20 mm for all the cases under consideration. For both double-layer and triple-layer MPPs, the experimental results have similar trends, as predicted by Maa’s theory and FE simulations when the inter-layer distance is varied, while the absorption performances in the experiments are found to be more effective with higher absorption coefficients in wider frequency ranges.

The results also show that the increase of the inter-layer distance shifts the peaks on the absorption curves to a slightly lower frequency range for both double-layer and triple-layer MPPs. The air between the panels plays the role of the acoustic spring, and, thus, has the effect on tuning the bandwidth of the absorption coefficient. The first peaks on the curves are not significantly affected by the change of the inter-layer distance. However, as the inter-layer distances increase, the values of the second peak on the curve of the double-layer MPPs decrease, while the position of the second peak on the curve of the triple-layer MPPs shifts to a lower frequency range with no significant change in absorption coefficient values, which is shown in Figure 8. The value of the third peak on the curve of the triple-layer MPP drops significantly when the inter-layer distance *h*_1_ increases from 30 mm to 50 mm.

For the implementation of MPPs, the distance between the acoustic devices and the wall is required to be constant, which varies the number of layers. The inter-layer distances between the panels is more feasible than adjusting the air gap distances only.

### 3.4. Structural Optimization for Sound Absorption

In Figure 6, Figure 7 and Figure 8, it is clear that the area under the curves depends on both the absorption coefficients and the frequency bandwidth, i.e., larger coefficients and a wider bandwidth give rise to a larger area. Therefore, the optimization of the sound absorption at the target frequency range can be presented by the maximization of the area under the sound absorption curve, which is defined as the frequency coverage in this study.

The main target frequency range in this study is set as 800–1200 Hz since most of the frequencies of the traffic noises are within this range [40]. The sound absorption at other frequencies can also be reached at the same time as multiple peaks with large absorption coefficients achieved using the multi-layer MPPs.

The space constraints should also be considered in designing the MPP structures for their installations on the buildings, in the rooms, or in the small structures including the ducts. The total length *L* of the MPP is determined by the construction requirement. At a certain *L*, the optimized geometric parameters of the multi-layer MPP structures to achieve the largest frequency coverage at the target frequency range can be predicted, according to Maa’s theory, which has been proven to be reliable when compared with the FEM and experimental results. In this study, *L* varied from 10 mm to 200 mm with an interval of 10 mm. The panel thickness *t*, perforation diameter *d*, and perforation ratio *p* of the panels are kept as 1 mm, 0.9 mm, and 1.0%, respectively.

**Figure 9:**
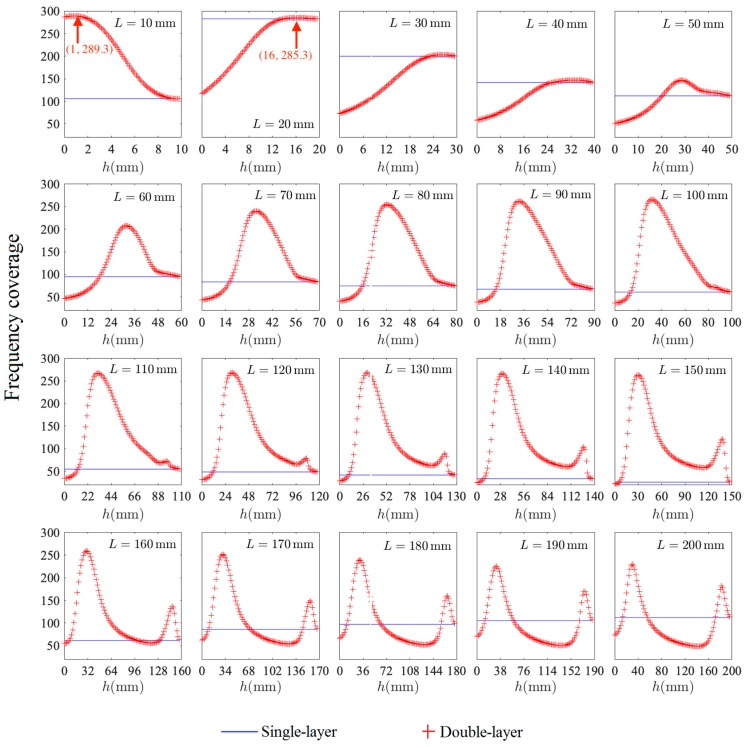
The frequency coverages of the single-layer and double-layer MPPs at each *L* varying from 10 mm to 200 mm.

The frequency coverages of the single-layer and double-layer MPPs with *L* varying from 10 mm to 200 mm are shown in Figure 9. The air gap distance of the single-layer MPP is fixed as *L* (*t* = 1 mm is ignored), and the largest value of the frequency coverage is reached at *L* = 20 mm. For the double-layer MPP, the sound frequency coverage at each *L* changes as the inter-layer distance *h* changes from 0 to *L*, and the peak value is reached at a certain value of *h*. The largest peak value of the frequency coverage of double-layer MPP is reached at *L* = 10 mm and *h* = 1 mm, and the second-largest frequency coverage is reached at *L* = 20 mm and *h* = 16.2 mm.

**Figure 10:**
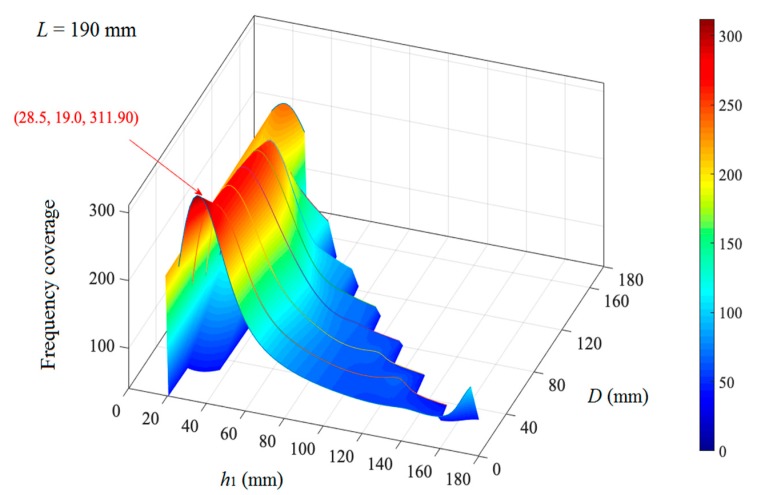
The frequency coverage of the triple-layer MPPs at *L* = 190 mm.

Similarly, the frequency coverages with a peak value for the triple-layer MPPs at each *L* are calculated. Among all the peak values of the frequency coverages at different *L*, the largest peak value is reached at *L* = 190 mm. The frequency coverages of the triple-layer MPPs with varying inter-layer distance *h*_1_ and air gap distance at *L* = 190 mm is shown in Figure 10. The largest frequency coverage is achieved at *h*_1_ = 28.5 mm and *D* = 19 mm.

**Figure 11:**
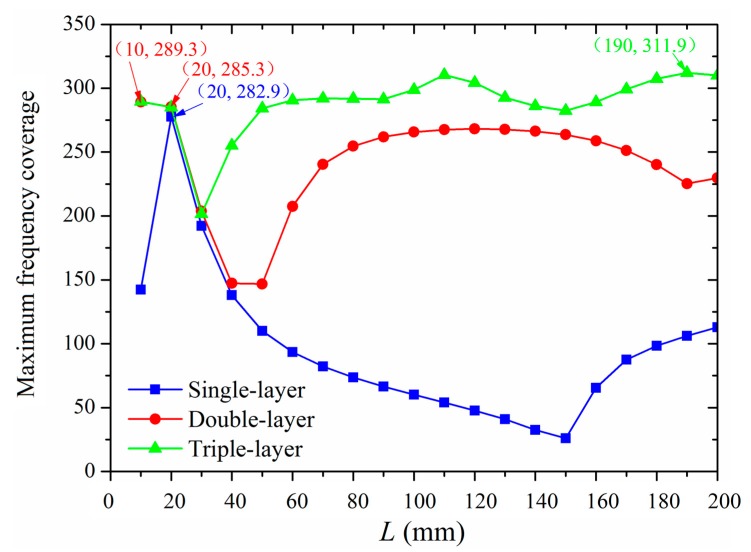
The maximum frequency coverage of single-layer, double-layer, and triple-layer MPPs at a different *L*.

The peak values of the frequency coverages of the double-layer and triple-layer MPPs are calculated and compared with the values of the single-layer MPP at each *L* in Figure 11. The largest frequency coverage achieved by a single-layer MPP is found to be 282.9 at *L* = 20 mm. For the double-layer MPP, the largest frequency coverage is observed with *L* = 10 mm and *h* = 1 mm (shown in Figure 9), which is not feasible to manufacture and install in the real-life application. Thus, the optimized double-layer MPP is designed at *L* = 20 mm and *h* = 16.2 mm when the maximum frequency coverage is at the second-highest value of 285.3 (shown in Figure 9). For the triple-layer MPP, at *L* = 190 mm, *h*_1_ = 28.5 mm, and *D* = 19 mm, the frequency coverage can reach up to 311.9 (shown in Figure 10), which is the largest among the single-layer and multi-layer structures with *L* varying from 10 mm to 200 mm.

**Figure 12:**
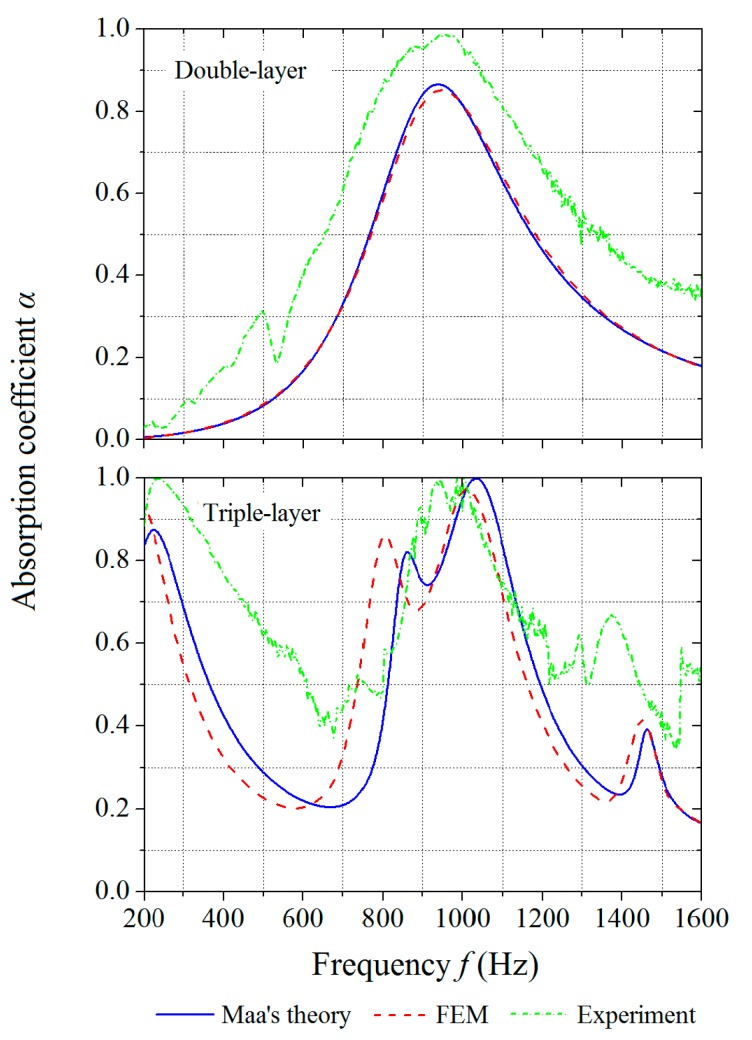
Absorption coefficients of optimized MPPs obtained by theoretical predictions, numerical simulations, and experiments: (**top**) double-layer MPP and (**bottom**) triple-layer MPP.

The optimized parameters for the double-layer MPP are found to be *h* = 16.2 mm and *D* = 3.8 mm, while the optimized parameters for the triple-layer MPP are *h*_1_ = 28.5 mm, *h*_2_ = 142.5 mm, and *D* = 19 mm. With the designed parameters, the sound absorption of the structures for the frequency range of 800–1200 Hz is optimized by the maximization of the frequency coverage, which is the area under the absorption curve. Figure 12 shows the absorption coefficients of the optimized structures by Maa’s theory, FE simulations, and experiments. The frequency coverage of the optimized double-layer MPP by experiment is 308.9, which is 8.3% larger than the theoretical prediction. The frequency coverage of the optimized triple-layer MPP by experiment is 319.3, which is 2.4% larger than the theoretical prediction. In this case, the absorption curve of the double-layer MPP only has one peak, while the absorption curve of the triple-layer MPP has three peaks with large absorption coefficients that indicate effective acoustic absorptions at multiple frequency ranges by the optimized structural design.

The frequency coverage can be maximized for designing MPPs to have highly effective sound absorptions in certain frequency ranges. The geometric parameters of an MPP can be determined by the developed optimization method in different target ranges of frequencies, according to application requirements. The optimization method by the sound frequency coverage can also be applied in a wider range of acoustic damping devices for effective sound absorptions in certain frequency ranges.

## 4. Conclusions

The advanced 3D printing technique, called SLS, was introduced to manufacture the MPP structures for acoustic absorption. The feasibility of using SLS and polymer materials to produce multi-layer acoustic designs at a high manufacturing convenience and a relatively low prototype cost was revealed. The performances of the multi-layer acoustic structures were evaluated by Maa’s theory, FE simulations, and experiments in a wide frequency range. The frequency ranges were tuneable by adjusting the structural parameters such as the number of layers, the air gap distance, and the inter-layer distance. The developed FE model was proved to be feasible for the numerical simulation of the multi-layer acoustic structures. An optimization method that maximizes the area under the sound absorption curve was introduced for the acoustic structures with the most effective sound absorption in target frequency ranges.

## Figures and Tables

**Table 1:** Geometric parameters of the tested specimens.

Sample Number	Number of Layers	Inter-Layer Distances
MPP_1	1	NA
MPP_2	2	*h* = 30 mm
MPP_3	2	*h* = 40 mm
MPP_4	2	*h* = 50 mm
MPP_5	3	*h*_1_ = 30 mm, *h*_2_ = 40 mm
MPP_6	3	*h*_1_ = 40 mm, *h*_2_ = 40 mm
MPP_7	3	*h*_1_ = 50 mm, *h*_2_ = 40 mm

**Table 2:** Mechanical and viscosity properties of PA12.

Tensile Modulus (MPa)	Tensile Strength (MPa)	Toughness (J/mm^3^)	Elongation at Break (%)	Viscosity (Pa·s) at 200 °C
1291 (±12.1)	44 (±1.3)	10.18 (±0.9)	24 (±0.8)	2616 (±99)
